# Differential Transcriptional Activation of Genes Encoding Soluble Methane Monooxygenase in a Facultative Versus an Obligate Methanotroph

**DOI:** 10.3390/microorganisms6010020

**Published:** 2018-03-06

**Authors:** Angela V. Smirnova, Peter F. Dunfield

**Affiliations:** Department of Biological Sciences, University of Calgary, 2500 University Drive NW, Calgary, AB T2N 1N4, Canada; avsmyrno@ucalgary.ca

**Keywords:** transcriptional regulation, methane oxidation, facultative methanotroph, obligate methanotroph, *Methylocella*

## Abstract

Methanotrophs are a specialized group of bacteria that can utilize methane (CH_4_) as a sole energy source. A key enzyme responsible for methane oxidation is methane monooxygenase (MMO), of either a soluble, cytoplasmic type (sMMO), or a particulate, membrane-bound type (pMMO). *Methylocella* *silvestris* BL2 and *Methyloferula* *stellata* AR4 are closely related methanotroph species that oxidize methane via sMMO only. However, *Methyloferula* *stellata* is an obligate methanotroph, while *Methylocella* *silvestris* is a facultative methanotroph able to grow on several multicarbon substrates in addition to methane. We constructed transcriptional fusions of the *mmo* promoters of *Methyloferula* *stellata* and *Methylocella* *silvestris* to a promoterless *gfp* in order to compare their transcriptional regulation in response to different growth substrates, in the genetic background of both organisms. The following patterns were observed: (1) The *mmo* promoter of the facultative methanotroph *Methylocella silvestris* was either transcriptionally downregulated or repressed by any growth substrate other than methane in the genetic background of *Methylocella* *silvetris*; (2) Growth on methane alone upregulated the *mmo* promoter of *Methylocella* *silvetris* in its native background but not in the obligate methanotroph *Methyloferula* *stellata*; (3) The *mmo* promoter of *Methyloferula* *stellata* was constitutive in both organisms regardless of the growth substrate, but with much lower promoter activity than the *mmo* promoter of *Methylocella* *silvetris*. These results support a conclusion that a different mode of transcriptional regulation of sMMO contributes to the facultative lifestyle of *Methylocella* *silvetris* compared to the obligate methanotroph *Methyloferula* *stellata*.

## 1. Introduction

Ever since the first methanotroph was described a century ago by Söhngen [1], it has been known that methanotrophs are actively involved in a global cycle of methane [2]. Aerobic methanotrophs inhabit diverse ecosystems where an anoxic/oxic interfaces exist, and oxidize anaerobically produced methane using O_2_ as an electron acceptor. The enzyme responsible for the initial step of methane oxidation is methane monooxygenase (MMO), which exists in two evolutionarily unrelated forms: a soluble, cytoplasmic form (sMMO), and a particulate, membrane-bound form (pMMO). Phylogenetically, known aerobic methanotrophs belong to the proteobacterial classes *Alphaproteobacteria* and *Gammaproteobacteria* [3], the phylum *Verrucomicrobia* [4,5,6,7,8,9] and the candidate phylum NC10 [10]. The *Alphaproteobacteria* methanotrophs are further divided into two families, *Methylocystaceae* and *Beijerinckiaceae* [11]. The *Beijerinckiaceae* family encompasses species with diverse phenotypes, including versatile chemoorganotrophs, phototrophs, obligate methanotrophs, facultative methylotrophs and methanotrophs [12]. The methanotrophs in this family include the only two genera known to use the sMMO enzyme exclusively to activate methane: *Methylocella* and *Methyloferula* [13,14]. All other methanotrophs possess just pMMO, or both pMMO and sMMO.

*Methyloferula stellata* AR4 is an obligate methanotroph, capable of growing on the C1 compounds methane or methanol as sole substrates [14,15]. In contrast, *Methylocella silvestris* BL2 was the first documented facultative methanotroph able to grow on multicarbon compounds in addition to methane. In terms of growth substrates, it is by far the most versatile methanotroph yet discovered [16], growing on C1 compounds (methane, methanol, formate, and methylamine) as well as organic acids (acetate, pyruvate, succinate, malate, gluconate, and propionate), alcohols (ethanol, 2-propanol, 1,2-propanediol, glycerol), short chain alkanes (ethane, propane), acetone, and methyl acetate [17,18,19]. Facultative methanotrophy, the ability to grow on substrates besides methane and related C1 compounds, has now been demonstrated in a few other (pMMO-using) methanotrophs [20,21,22,23]. However, the range of growth substrates for these other facultative methanotrophs is much narrower than for *Methylocella*, generally limited to acetate, ethanol, and H_2_. 

In *Methylocella silvestris* BL2, the sMMO is repressed at the transcriptional level in the presence of alternative substrates like acetate [17,19]. In contrast, other facultative methanotrophs like *Methylocapsa aurea* and *Methylocystis* sp. H2 grow more efficiently on methane rather than acetate and/or ethanol. These other methanotrophs utilize a pMMO to convert methane to methanol and possess a well-developed intracytoplasmic membrane (ICM) in which pMMO is bound. Interestingly, *Methylocystis* strain H2, which has functional genes for both sMMO and pMMO, was shown to express only pMMO regardless of tested growth conditions. Moreover, pMMO was expressed constitutively in the facultative *Methylocystis* strains H2 and SB2, even in the presence of alternative substrates [21]. *Methylocella* and *Methyloferula* lack pMMO and extensive ICM. 

The obligate methanotroph *Methyloferula stellata* AR4 is closely related to *Methylocella silvestris* BL2 (97.1% identity of 16S ribosomal RNA (rRNA) genes). A recently published draft genome of *Methyloferula stellata* AR4 revealed little difference in functional genes involved in methane metabolism compared to *Methylocella silvestris* BL2 [14]. Both use only sMMO to convert methane to methanol, plus similar pathways for further processing of methanol. However, *Methyloferula stellata* AR4 is unable to grow on any of the alternative, non-C1 substrates that *Methylocella silvestris* BL2 uses. Therefore, we compared transcriptional activities of the *mmo* promoters in the two organisms. Transcriptional fusions of the promoters to a promoterless reporter gene, *gfp*, and their responses to growth on different substrates were tested in the genetic backgrounds of both organisms. 

## 2. Materials and Methods

### 2.1. Bacterial Strains and Growth Conditions

*Methylocella silvestris* BL2 and *Methyloferula stellata* AR4 were cultivated in DAMS (pH 5.8) and MM2 (pH 4.8–5.2) media, respectively, as described previously [13,15]. *M. silvestris* BL2 was maintained on DAMS agar plates, whereas *M. stellata* AR4 was maintained on MM2 plates containing Phytagel as a solidifying agent. Plates were incubated at 25 °C in an anaerobic jar (Oxoid, Nepean, ON, Canada) containing 20% (*v*/*v*) methane in air. Routinely, 30 mL of liquid cultures were grown in serum bottles (120 mL) sealed with butyl rubber stoppers and supplemented with either 20% (*v*/*v*) methane in the headspace or 0.5% (*v*/*v*) methanol in a medium, and incubated at 25 °C on a shaker at 120 rpm. *M. silvestris* BL2 cultures were also cultivated in 30 mL of DAMS (Diluted ammonium mineral salts) medium containing 5 mM sodium acetate as a growth substrate. For growth and methane consumption experiments, cells were cultivated in 250 mL or 1 L bottles sealed with GL45 chlorbutyl septa (Glasgerätebau Ochs, Bovenden, Niedersachsen, Germany) and open-top caps (VWR, Edmonton, AB, Canada). Growth of cells was monitored via optical density at 600 nm using an Ultrospec spectrophotometer (GE Healthcare Life Sciences, Mississauga, ON, Canada). A decrease of methane in the headspace of growth vials was quantified using an SRI 8610C Gas chromatograph equipped with a HayeSep-D column coupled to a flame ionization detector (FID) (column T 100 °C; detector T 300 °C; N_2_ as carrier gas). A certified 0.5% (*v*/*v*) methane in air mixture was used as a standard (Praxair, Danbury, CT, USA).

*E.coli* DH5α strain was used to propagate plasmids for cloning in LB medium. Kanamycin (20 mg/L) and ampicillin (100 mg/L) were used to maintain plasmids in *E. coli* DH5α, *M. silvestris* BL2, and *M. stellata* AR4.

### 2.2. DNA Manipulations, Plasmid Construction

FastDNA Spin Kit (MP Biomedicals, Solon, OH, USA) and EZ-10 Spin Column Plasmid Prep kits (BioBasic Inc., Markham, ON, Canada) were used to isolate genomic and plasmid DNA, respectively, according to the manufacturer’s instructions. Polymerase chain reaction (PCR) primers were designed using the genomes of *M. silvestris* BL2 and *M. stellata* AR4 (Accession NC_011666 and NZ_ARWA00000000, respectively) and Vector NTI software (ThermoFischer Scientific, Waltham, MA, USA). Primers were synthesized by ThermoFischer Scientific (USA). The primers sMMO_EcoRI_f, 5′-ACTGAATTCAGCCCGTTGTCGCTTTGATA-3′, and sMMO_SacI_rev, 5-ACTGAGCTCATGTCTCCTCCTTGGTGCTC-3′, were used to amplify a 1080 bp fragment containing a promoter region of sMMO upstream of the *mmoX* gene of *M. silvestris* BL2. The primers Pmmo AR4_EcoRI_F, 5′-ACTGAATTCGCGAAATACGATACGCCGAC-3′ and Pmmo AR4_SacI_R, 5′-ACTGAGCTCTCATCGCTCTGGTGCTTTGA-3′ were used to amplify an 889 bp fragment containing a promoter region of sMMO upstream of the *mmoX* gene of *M. stellata* AR4. PCRs were performed with Phusion proofreading DNA polymerase (Bioline, Taunton, MA, USA) under conditions recommended by the manufacturer. A two-step PCR procedure was used for cycling. Initially, DNA was denaturated at 98 °C for 3 min, followed by 35 amplification cycles (denaturation at 98 °C for 20 s, annealing and extension at 72 °C for 40 s), plus a final extension at 72 °C for 7 min. PCR products were purified using an EZ-10 Spin Column PCR Products Purification Kit (BioBasic Inc., Canada), digested with *Eco*RI and *Sac*I (ThermoFischer Scientific, USA) and ligated into pMHA200 [19]. Promoter fusions to *gfp* in the resulting plasmids, pAS31 and pAS50, were confirmed by Sanger sequencing (Eurofins MWG Operon, Louisville, KY, USA) with forward and reverse primers: seq gfp 5′-AACAATTTCACACAGGAAAC-3′, seq TF_gfpBL2 5′-ACAACAGAGCTGCCGCACTG-3′, seq TF_gfpAR4 5′-ACGACTATCGCCCCAATTTG-3′.

### 2.3. Preparation of Competent Cells and Electroporation into M. silvestris BL2 and M. stellata AR4

Cells were grown in batch culture. A stock culture was added to either 500 mL DAMS medium for *M. silvestris* or 500 mL of MM2 medium for *M. stellata* in 1 L bottles. Methane (20% *v*/*v*) was added to the headspaces. Altogether, six 1 L bottles containing bacterial inoculum were incubated at 25 °C, shaken at 120 rpm, for either 9 days (*M. silvestris* BL2) or 21 days (*M. stellata* AR4). Exponentially growing cells were cooled on ice for 15 min, harvested by centrifugation (6000× *g* for 15 min at 4 °C), washed twice in ice-cold water (4 °C), and finally resuspended in 10 mL of 10% glycerol (*m*/*v*). Finally, 300–400 µL of suspended cells were aliquoted in 1.5 mL centrifuge tubes, frozen in liquid nitrogen, and stored at −80 °C until used. For electroporation, 200 µL of competent cell suspension was gently mixed with 400–500 ng of plasmid DNA and transferred to a 2-mm cooled electroporation cuvette (Eppendorf Canada, Mississauga, ON, Canada). Electroporation was performed at 2.2 kV for 4–5 ms, using an Eppendorf Eporator (Eppendorf Canada, Mississauga, ON, Canada). Cells were washed from the cuvette with either 1 mL of DAMS containing 0.5% ethanol for *M. silvestris* or 1 mL of MM2 containing 0.5% methanol for *M. stellata*. Cells were allowed to recover overnight at 25 °C with shaking at 120 rpm. Cells were pelleted by centrifugation (1500× *g*, 8 min) at room temperature and finally resuspended in 250 µL of DAMS or MM2 medium before plating out either on DAMS-agar plates for *M. silvestris* or on MM2-phytagel plates for *M. stellata*, with kanamycin as an antibiotic for positive selection. After incubation for three weeks in an atmosphere containing methane, kanamycin-resistant colonies appeared. A few colonies were transferred twice onto new plates with kanamycin and cells were then were transferred to liquid medium containing kanamycin, supplied with 20% (*v*/*v*) methane in the headspace. If transformants could not grow on liquid medium with kanamycin, they were discarded. After a few passages in liquid medium with kanamycin, transformants were subjected to microscopy studies to analyze their fluorescence. Genomic DNA was isolated from one transformant for each transcriptional fusion in the respective host. A 1.2-kb fragment for the *M. silvestris* promoter and a 1-kb fragment for the *M. stellata* promoter were amplified from genomic DNAs using the sequencing primers seq gfp, seq TF_gfpBL2, seq TF_gfpAR4 (see above), to prove the presence of transcriptional fusions in the respective host.

### 2.4. Fluorescence Microscopic Detection of GFP-Labelled Cells

To assess promoter activities, we transferred colonies of the positive transformants into liquid media with methane (20% *v*/*v*), methanol (0.5% *v*/*v*), or acetate (5 mM) as the sole energy source. Six ml of bacterial cultures after 3–4 weeks of growth were harvested by centrifugation, resuspended in 25 µL of PBS buffer, and 10 µL of the cell suspension was added to a cover slip. A drop of Prolong Diamond Antifade Mountant (ThermoFischer Scientific, USA) was added to a glass slide. Subsequently, the cover slip was placed sample-side down onto the mountant on the glass slide. To cure, the glass slide was incubated at room temperature for 24 hours. Fluorescence of single cells was detected using an Olympus BX51 fluorescence microscope (Olympus Canada Inc., Richmond Hill, ON, Canada) with an excitation filter at 460–490 nm and an emission filter at wavelengths >520 nm, using a 100× oil immersion objective. Image-Pro Express (version 6.0) software was used to view images.

### 2.5. Flow Cytometry Analysis of Promoter Fusion to gfp

To quantify green fluorescent protein (GFP)-fluorescent cells, the cell suspension in PBS buffer that was used for fluorescence microscopy was diluted to 2–3 mL using sheath fluid (BD Biosciences, Mississauga, ON, Canada) to adjust the concentration of cells to 10^8^ cells/mL. A proper dilution to 10^8^ cells/mL was achieved by measuring optical density at 600 nm. A sample was injected into a FACScalibur (BD Biosciences, USA) flow cytometer equipped with a 15-mW air-cooled argon-ion laser as the excitation light source (488 nm). Fluorescence in the range of 515–545 nm (FL1) was detected via a fluorescence detector set at a photomultiplier tube range of either 525 V (for *M. silvestris*) or 580 V (for *M. stellata*) with logarithmic gain. Forward scatter (FSC) was collected by a diode with an amplification factor of E00 and processed in logarithmic gain. Side scatter (SSC) was detected in logarithmic gain by a photomultiplier tube set at 356 V. Each sample was collected for 60 s at high flow rate. A total of 10,000 events were collected for each measurement. The BD CellQuest Pro software was used to generate histograms, plots and perform analysis (BD Biosciences, Mississauga, ON, Canada).

### 2.6. Quantitative Measurements of Promoter Activities

To quantify promoter activities, replicate samples of harvested bacterial cultures were resuspended in 200 µL of PBS buffer. Cell suspensions were placed into wells of a black microtiter plate. Subsequently, the emission of GFP-labeled cells was recorded on an EnSpire Multimode Plate Reader (PerkinElmer, Woodbridge, ON, Canada). All fluorescence values were normalized by total cell protein concentrations. First, cells suspensions were transferred into 1.5 mL tubes and processed through 5 times freeze-thaw cycles (from −80 °C to room temperature). Second, proteins were precipitated by adding an equal volume of cold (4 °C) 10% trichloroacetic acid (TCA) and denatured by boiling for 5–10 min. After centrifugation for 5 min, the protein pellet was dissolved in 100 µL of 1 M NaOH and diluted to 1 mL with 900 µL ddH_2_O. Finally, the protein concentration was measured using a Qubit protein assay kit and Qubit fluorimeter (ThermoFischer Scientific, Waltham, MA, USA). 

For *M. silvestris* cells, fluorescence was tested in a single trial with 3–5 replicate cultures. For *M. stellata*, because the differences in fluorescence were noticeably smaller, the fluorescence was tested in 3 separate trials with 2–4 replicate cultures each. Fluorescence of control *M. stellata* cultures (i.e., cultures with *gfp* fused to no promoter) varied between the different trials. Therefore, for statistical testing, data were blocked by the trial, although only the overall averages are shown in the relevant figures. A General Linear Model (analysis of variance; ANOVA) was performed using Systat v.13 (Systat Software Inc., San Jose, CA, USA) with: Blocks (four trials), Host organisms (two levels), Promoters (three levels; two promoters + one control with no promoter); and Growth substrates (three levels). The design was incomplete because some treatments were impossible (e.g., growth on acetate in *M. stellata*). Each treatment combination tested contained at least three replicates.

## 3. Results

### 3.1. Growth of M. silvestris and M. stellata on Methane, and Effect of Cerium (III)

We compared growth of *M. silvestris* and *M. stellata* on methane and quantified growth and methane consumption by both organisms. Monitoring cell density for triplicate cultures of *M. silvestris* and *M. stellata* demonstrated that *M. silvestris* grew more rapidly under these conditions, with a faster rate of methane depletion in the culture vials (Figure S1a) and an higher growth rate constant (µ) of 0.0018 ± 0.0002 h^−1^ (mean ± 1SEM), compared to 0.0011 ± 0.0002 h^−1^ for *M. stellata*. Previously reported values for growth rates were 0.0138 and 0.005 h^−1^ for *M. silvestris* and *M. stellata*, respectively [13,15]. 

Because methane was the only substrate available to generate energy and cell mass in this experiment, a difference in growth rates and rates of methane consumption could be explained in terms of differential activity and function of sMMO enzymes between the two organisms. For instance, lack of a component that promotes sMMO activity could result in decreased rates of methane consumption. A recent report indicated that depletion of light rare earth elements (REE) co-occurred with methane consumption during the *Deepwater Horizon* well blowout in the ocean [24]. Moreover, the light REE (La, Ce, Pr, and Nd) are essential for at least some methanotrophs, being components that mediate a transcriptional switch between two types of methanol dehydrogenase (MDH): MxaF and XoxF [25,26]. It was also demonstrated that REE were essential as a cofactor in the XoxF enzyme of the verrucomicrobial methanotroph, *Methylacidiphilum fumariolicum* SolV [27]. Therefore, we tested an effect of cerium (III) addition on growth and methane consumption in *M. silvestris* and *M. stellata* cultures. Previous studies used the concentration of 25 µM of cerium (III) chloride [26,28]. In these studies, the effect of cerium on transcription of *mxaF* and *xoxF* genes was obvious. Therefore, we added 25 µM of cerium (III) chloride to the respective growth media for *M. silvestris* and *M. stellata* and subsequently monitored cell density and methane consumption for both organisms (Figure S1b). The addition of cerium (III) had no positive effect on growth of *M. stellata*. The growth rate of *M. stellata* upon cerium (III) addition was 0.00096 ± 0.000 h^−1^ vs. 0.0011 ± 0.0002 h^−1^ without cerium (III). In *M. silvestris*, the addition of cerium (III) resulted in changes in the appearance of cultures. Cells aggregated to form large flakes or other filamentous structures when cerium (III) was added (Figure S2). *Methylocella silvestris* produces extensive Exopolysaccharides (EPS), particularly when grown on alternative substrates rather than methane. Nevertheless, we had never observed such filamentous structures. Therefore, the experiment was repeated and showed the same phenotype when cerium (III) was added. Figure S2 shows bottles after cultures were shaken in order to measure optical density (OD)_600nm_. These morphologically different cultures did not allow monitoring cell density properly via OD for calculating growth rates of *M*. *silvestris*. However, a decrease in the rate of methane depletion in the culture vessels indicated a slower growth rate (Figure S1b). Cerium (III) therefore has different effects on the two studied organisms. We intended to test whether cerium (III) has an upregulating (positive) effect on methane oxidation and the *mmo* promoter activity of either culture. However, there was no effect for *M*. *stellata* and a negative effect for *M*. *silvestris* cultures (data not shown).

### 3.2. Comparative Genomic Analysis of sMMO Encoding Genes

**Figure 1:**
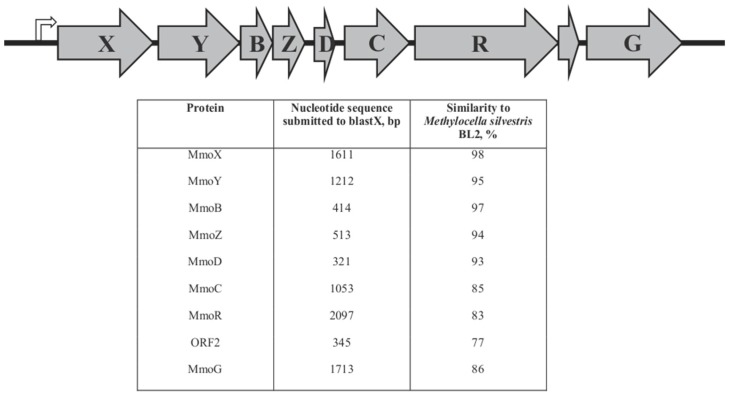
Identical organization of the *mmo* operons in the genomes of *M. silvestris* BL2 and *M. stellata* AR4, with amino acid similarities (%) of the homologues in the two organisms calculated via BLASTX.

In *M. silvestris*, sMMO is encoded by the genes *mmoX*, *Y*, *B*, *Z*, *C*, *R*, *G*, and a gene designated as ORF2, which are transcribed as an operon from a σ^54^-dependent promoter [19]. We compared genetic organization of *mmo* genes involved in methane oxidation in *M. silvestris* BL2 and *M. stellata* AR4 (Accession NC_011666 and NZ_ARWA00000000). With respect to gene order and number, *mmo* gene clusters are identical in these organisms (Figure 1). According to the results of reciprocal BLASTX searches, proteins MmoX, Y, Z, which are α, β, and γ subunits of the hydrolase component, show a high degree of amino acid similarity (94–98%). MmoB and MmoD, which play roles in coupling of other structural proteins and correct assembly of the unique di-iron centre of the sMMO enzyme, respectively, also share a high degree of similarity (97% and 93%, respectively) (Figure 1). Similarities for the reductase MmoC, ORF2, regulatory protein, MmoR, and the putative GroEL-like chaperon MmoG, were somewhat lower (77–86%). MmoR is the only transcriptional regulator in the *mmo* operon. It belongs to the family of the σ^54^-dependent transcriptional regulators and uses adnosine triphosphate (ATP) binding and hydrolysis to catalyze the formation of open promoter complexes for transcriptional initiation [29]. This was confirmed by an analysis of the secondary structure of MmoR proteins of *M. silvetris* and *M. stellata* using online resources [30]. Analyses of domain organization also revealed that the N-termini of MmoR proteins from both organisms contain GAF (cGMP-specific phosphodiesterases, adenylyl cyclases and FhlA) domains, which are not found in the model methanotrophs *Methylosinus trichosporium* OB3b or *Methylococcus capsulatus* Bath. Therefore, both primary and secondary structures of MmoR indicate functional similarities between these regulators in the two model organisms.

### 3.3. Trascriptional Analyses of sMMO-Encoding Genes Using Green Fluorescent Protein (GFP) as a Reporter: Qualitative Analyses

In order to compare promoter activities for genes encoding sMMO in the genetic background of either organism, we generated transcriptional fusions of the *mmo* promoter of *M. silvestris* and of the *mmo* promoter of *M. stellata* to a promoterless *gfp* in plasmid pMHA200 [19]. This resulted in two plasmids, pAS31 (*M. silvestris*) and pAS50 (*M. stellata*), respectively. Plasmids were transferred into *M. silvestris* and *M. stellata* by electroporation, resulting in four combinations: (1) *M. silvestris gfp*-fusion promoter in *M. silvestris*; (2) *M. silvestris gfp*-fusion promoter in *M. stellata*; (3) *M. stellata gfp*-fusion promoter in *M. stellata*; and (4) *M. stellata gfp*-fusion promoter in *M. silvestris*. Plasmid pMHA200, which carries a promoterless *gfp*, was electroporated into both organisms to use as a negative control.

**Figure 2:**
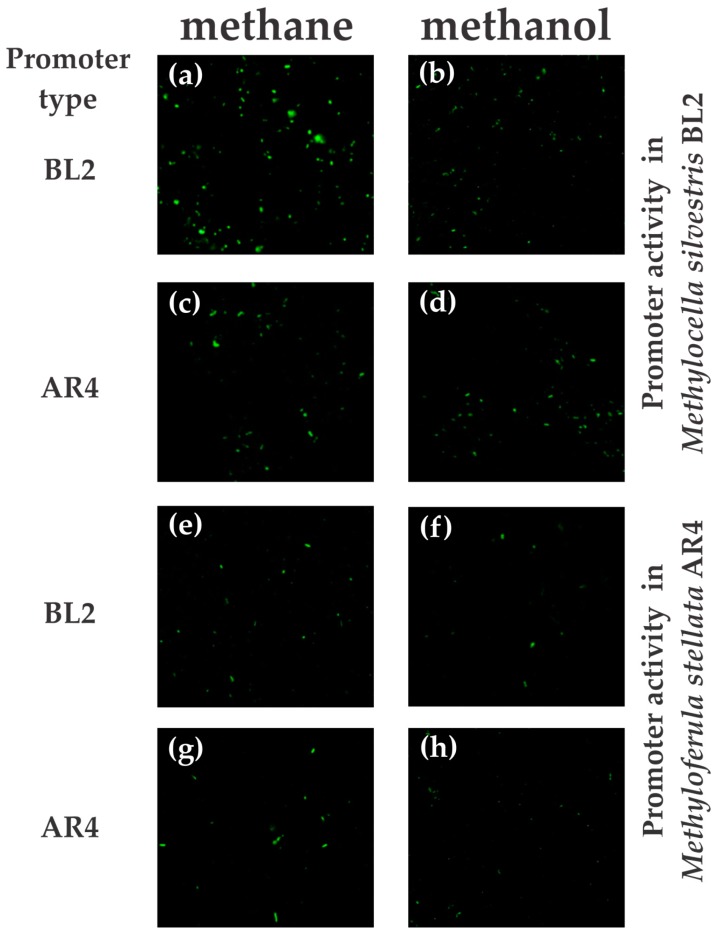
Fluorescence microscopy images of *M. silvestris* BL2 and *M. stellata* AR4 cells carrying transcriptional fusions of a promoterless *gfp* to the respective *mmo* promoters and growing on either methane or methanol. Panels (**a**,**b**) show the *mmo* promoter of *M. silvestris* BL2 in *Methylocella*; panels (**c**,**d**) show the *mmo* promoter of *M. stellata* AR4 in *Methylocella*; panels (**e**,**f**) show the *mmo* promoter of *M. silvestris* BL2 in *Methyloferula*, and panels (**g**,**h**) show the *mmo* promoter of *M. stellata* AR4 in *Methyloferula*. The images are representative of one of three independent experiments, and each treatment used similar cell densities.

Firstly, we reproduced results of a previous study [19], which investigated the transcription of the *mmo* promoter of *M. silvestris* with respect to growth on either methane or acetate. In addition to methane and acetate, we also studied an effect of growth on methanol on the transcriptional activation of the *mmo* promoter. Fluorescence images were taken of *M. silvestris* cells carrying the native *mmo* promoter fused to *gfp*. Cells cultivated on methane (Figure 2a) showed a higher level of GFP expression compared to cells cultivated on methanol (Figure 2b). The same inhibitory effect on *mmo* expression was observed in acetate-grown cells compared to methane-grown cells (Figure S3a,b). Secondly, images were taken of *M. silvestris* cells carrying the *mmo* promoter of *M. stellata*, when cultivated on methane (Figure 2c), methanol (Figure 2d), and acetate (Figure S3c,d). Images showed low levels of GFP expression, which did not seem to be affected by the growth substrate provided. *M. silvestris* cells carrying control plasmid pMHA200 did not show any fluorescence (data not shown). Thirdly, we tested expression of both promoter constructs in *M. stellata*. In the genetic background of *M. stellata*, both *mmo* promoters showed low levels of GFP fluorescence on methane and methanol (Figure 2e–h). *M. stellata* cells carrying control plasmid pMHA200 did not show any fluorescence (data not shown). The only clearly visible effect of this experiment was therefore a higher activity of the *mmo* promoter of *M. silvestris*, in the genetic background of its native host, when growing on methane. All other treatments showed a lower activity.

**Figure 3:**
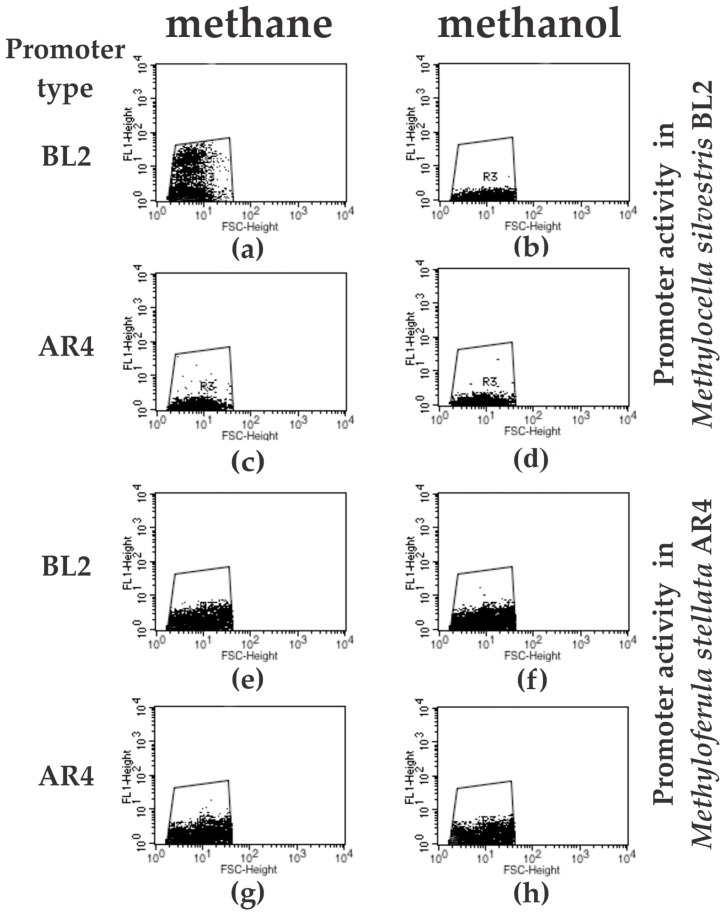
Flow cytometry analysis of *M. silvestris* BL2 and *M. stellata* AR4 cells carrying transcriptional fusions of a promoterless *gfp* to the respective *mmo* promoters and growing on either methane or methanol. Panels (**a**,**b**) show the *mmo* promoter of *M. silvestris* BL2 in *Methylocella*; panels (**c**,**d**) show the *mmo* promoter of *M. stellata* AR4 in *Methylocella*; panels (**e**,**f**) show the *mmo* promoter of *M. silvestris* BL2 in *Methyloferula*; and panels (**g**,**h**) show the *mmo* promoter of *M. stellata* AR4 in *Methyloferula*. The images are representative of one of three independent experiments. In each case 10,000 cells were counted.

Flow cytometry was used to verify the expression pattern observed above. Cell density of the cultures was adjusted to 10^8^ cells/mL, and GFP fluorescence was assessed for 10,000 cells of each treatment. As above, the largest ratio of strongly GFP-expressing cells was measured for *M. silvestris* carrying its native *mmo* promoter and supplied with methane (Figure 3a). Essentially no fluorescent cells were counted when this culture was grown on methanol (Figure 3b). A small number of fluorescent cells were counted for *M. silvestris* culture bearing a transcriptional fusion of the *mmo* promoter of *M. stellata* when cultured on either methane or methanol (Figure 3c,d). All *M. stellata* cells bearing transcriptional fusions of *gfp* to either *M. silvestris* BL2 or *M. stellata* AR4 promoters also showed a low level of fluorescence (Figure 3e–h).

### 3.4. Trascriptional Analyses of sMMO-Encoding Genes Using Green Fluorescent Protein (GFP) as a Reporter: Quantitative Analyses

To confirm the qualitative results derived from fluorescence microscopy and flow cytometry analysis, we also quantitatively measured promoter activities using a fluorescence microplate reader. All fluorescence values were normalized by total protein concentration. Significance was tested via a General Linear Model (GLM or ANOVA) and a post hoc Tukey’s Honestly Significant Difference Test, based on 3–5 replicate cultures per treatment. 

**Figure 4:**
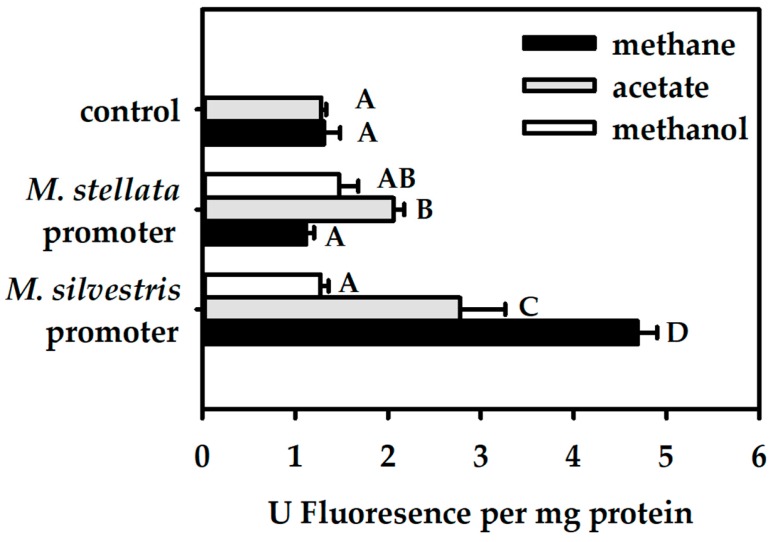
Quantitative measurements of fluorescence emitted by green fluorescent protein (GFP)-labeled cells normalized by total protein concentration, of *M. silvetris* BL2 cells growing on methane, methanol, or acetate. Emission was recorded for *M. silvestris* BL2 cells carrying transcriptional fusions of a promoterless *gfp* to either the *mmo* promoter of *M. silvestris*, or the *mmo* promoter of *M. stellata*. Cells carrying a *gfp* without any promoter fused to it were used as a control. Three to five replicates for each sample were used for measurements. Significant differences based on a General Linear Model and a post hoc Tukey’s Honestly Significant Difference Test (*p* < 0.05) for each treatment combination are indicated as letters next to each bar.

*M. silvestris* cells were cultivated on methane, methanol and acetate. Compared to methane, methanol and acetate both resulted in less transcription of the *M. silvestris mmo* promoter, with methanol showing stronger repression (Figure 4). The *mmo* promoter activity for methanol-fed cells was four times lower than for methane-fed cells. We conclude that growth on acetate downregulates the *mmo* promoter of *M. silvestris*, whereas growth on methanol leads to a near complete repression of transcriptional activation.

The substrate effect on *M. silvestris* cells containing the *M. stellata mmo* promoter was the opposite of that observed for the native *M. silvestris mmo* promoter (Figure 4). The slightly elevated but nevertheless significant expression levels above a negative control (cells carrying plasmid pMHA200) in at least one treatment (acetate) demonstrates that the construct was successful, and that the *mmo* promoter of *M. stellata* remained active in *Methylocella*, albeit at a low level. However, the *M. stellata* promoter was regulated in a dramatically different way from the *M. silvestris* promoter. The non-methane substrates not only did not repress the promoter activity, but actually stimulated it. The promoter may simply respond to cell metabolic status in some way.

**Figure 5:**
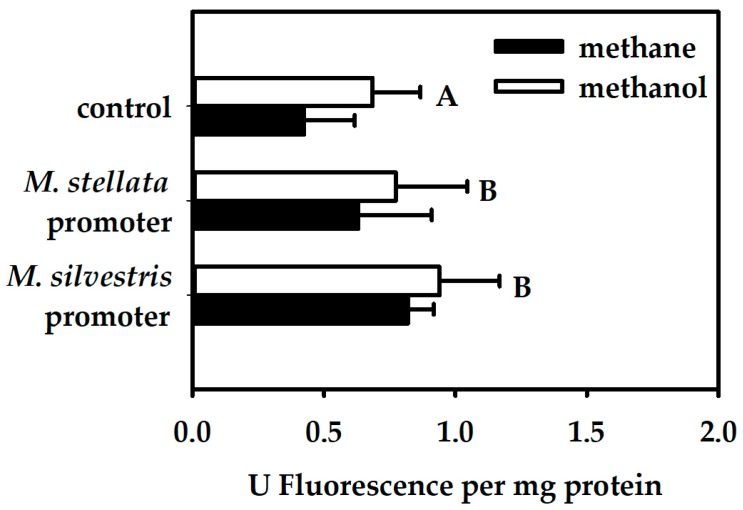
Quantitative measurements of fluorescence emitted by GFP-labelled cells normalized by total protein concentration, for *M. stellata* AR4 cells growing on either methane or methanol. Emission was recorded for *M. stellata* AR4 cells carrying transcriptional fusions of a promoterless *gfp* to either the *mmo* promoter of *M. silvestris*, or the *mmo* promoter of *M. stellata*. Cells carrying a *gfp* without any promoter fused to it were used as a control. Three replicates for each sample were used for measurements. Significance was tested using a General Linear Model and a post hoc Tukey’s Honestly Significant Difference Test. The overall effect of growth substrate was not significant in the GLM. Significant differences (*p* < 0.05) of the controls versus the two promoter constructs (pooled over both growth substrates) are indicated as letters.

When the respective promoter activities were measured in *M. stellata* cells cultivated on either methane or methanol, transcriptional activation was not significantly affected by growth substrate (GLM, *p* > 0.05). Expression levels for both promoters were low but each was significantly higher than the negative control cells carrying plasmid pMHA200 (Figure 5). Both the *mmo* promoter of *M. silvestris* and the *mmo* promoter of *M. stellata* were therefore active in *M. stellata*. However, the mode of regulation of the *M. silvestris* promoter was different-expression was not eliminated by methanol as it was in its native *M. silvestris*.

These quantitative effects observed by measuring whole-culture fluorescence could also be demonstrated by quantifying the flow cytometry data as follows: (1) Either *M. silvestris* or *M. stellata* cells carrying control plasmid pMHA200 (no promoter fused to *gfp*) were used to set up a fluorescence threshold (i.e., background fluorescence of the cells); (2) The number of events (the number of fluorescence cells) above the threshold for each treatment was counted using the BD CellQuest Pro software. We performed this quantitative estimation of fluorescent cells for *M. silvestris* cells growing on methane, acetate and methanol, and for *M. stellata* cells growing on methane and methanol. Results are presented in Supplemental Figure S4. Growth on methane yielded the largest number of fluorescent cells above the threshold for *M. silvestris* cells carrying its native *mmo* promoter (Figure S4a), whereas growth on acetate and methanol reduced the number of fluorescent cells. Growth on acetate yielded half as many fluorescent cells as compared to methane-fed cells. Methanol-fed cells showed the most dramatic reduction of the number of fluorescent cells (36 times less). Growth of *M. silvestris* cells carrying the *mmo* promoter of *M. stellata* yielded fewer fluorescent cells compared to its native *mmo* promoter regardless the growth substrate (Figure S4a). Nevertheless, it was again evident that the *mmo* promoter of *M. stellata* was transcriptionally active in *M. silvestris* cells, albeit at a low level. All *M. stellata* cells bearing transcriptional fusions of *gfp* to either *M. silvestris* or *M. stellata* promoters yielded a small quantity of fluorescent cells (Figure S4b). There was no obvious effect of growth substrate on the number of fluorescent cells.

In summary, the *mmo* promoter of *M. stellata* appeared to express GFP constitutively in its native organism and in its sister organism *M. silvestris*. This expression was always low and was either not affected or stimulated by growth on alternative substrates to methane. The activity of the *mmo* promoter of *M. silvestris* in its native organism was much higher in the presence of methane and was repressed to different extents by alternative substrates. However, the *M. silvestris mmo* promoter was much weaker in *M. stellata* growing on methane and was not negatively affected by methanol (the only alternative substrate that *M. stellata* will metabolize). These findings indicate that a different mode of sMMO gene regulation has been adopted by the facultative methanotroph *Methylocella silvestris* compared to the obligate methanotroph *Methyloferula stellata*.

### 3.5. Other Potential Cues for Transcription

We tested the effects of excess carbon dioxide (CO_2_, at 5–10% *v*/*v*) and cerium (III) (25 µM) on transcriptional activation of the *mmo* promoter of *M. stellata*, in order to determine if these environmental cues might enhance transcriptional activation. *M. stellata* cultures carrying the *mmo*-*gfp* transcriptional fusion and growing under elevated carbon dioxide did not show any increase in GFP expression (data not shown). Neither did we observe an effect on transcriptional activation upon addition of 25 µM cerium (III) chloride solution to *M. stellata* growing on either methane or methanol.

## 4. Discussion

In this study, we demonstrated that transcriptional activation of the *mmo* promoter in the facultative methanotroph *Methylocella silvestris* BL2 was dependent on the presence of methane as the growth substrate, whereas transcriptional activation of the *mmo* promoter in the obligate methanotroph *Methyloferula stellata* AR4, was constitutive regardless of the growth substrate. Methane dramatically enhances transcriptional activation of the *mmo* promoter in *M. silvestris*. Other growth substrates either downregulate or repress transcriptional activation of the *mmo* promoter. Interestingly, the *mmo* promoter of *M. stellata* was constitutively expressed either in the *M. silvestris* or *M. stellata* cells and the promoter activity was low regardless growth substrate. Even though *M. silvestris* and *M. stellata* are closely related organisms (97.1% identity of 16S rRNA genes), and their proteins involved in methane oxidation share high degrees of similarity, their mode of regulation of the *mmo* promoter is very different.

From previous studies [19,31,32] we know that the transcription of *mmo* genes in *Methylosinus trichosporium* OB3b, *Methylococcus capsulatus* Bath, and *Methylocella silvestris* BL2 is RpoN-dependent. Inactivation of *rpoN*, which encodes σ^54^ factor, resulted in abolition of *mmo* gene expression in *M. trichosporium* OB3b [32]. A-24-12 recognition motif (YTGGCACGrNNNTTGCW) characteristic of σ^54^-type promoters [29] can be identified upstream of every sequenced *mmoX* gene [19], including sequences in *M. silvestris* BL2 and *M. stellata* AR4. Unlike the major σ^70^-factor, the σ^54^-factor relies on a specialized class of transcriptional activators (enhancers), which uses ATP binding and hydrolysis to catalyze the formation of open promoter complexes for transcriptional initiation. In numerous cases such activators, for instance NtrC, are members of two-component signal transduction pathways, and require phosphorylation of an N-terminal receiver domain to ‘translate’ environmental cues into a coherent transcriptional activation of genes [29]. A gene encoding a σ^54^-dependent transcriptional activator, *mmoR*, is located in *mmo* operons of both *M. silvestris* and *M. stellata*. Homologues of *mmoR* are also located in the vicinity of the *mmo* operons in other methanotrophs [33]. Notably, a gene encoding a cognate histidine protein kinase, another member of two-component regulatory system, which is usually located adjacent to a response regulator, is not found in the sMMO-coding regions of the genomes of *M. silvestris* and *M*. *stellata*. The GasS/GacA two-component regulatory system provides an example where the *gacS* gene coding for a histidine kinase is physically unlinked on bacterial chromosome to the *gacA* gene encoding a response regulator [34]. However, a receiver domain, which contains a site being phosphorylated by a cognate histidine kinase, was not identified in MmoR proteins by a search of conserved domains in our study.

Although Theisen et al. [19] claimed that the conserved Asp residue (phosphorylation site) was found in an alignment of the MmoR sequences of *M. trichosporium*, *M. capsulatus*, *M. silvestris* and selected other σ^54^—transcriptional regulators, it is not certain that MmoR is activated via phosphorylation by a cognate histidine kinase, since if this were so then potential cognate histidine kinases should also be highly homologous and the mode of regulation of *mmo* genes should therefore been similar in all methanotrophs. Our result contradicts this assumption. Not all σ^54^—transcriptional regulators require phosphorylation to regulate transcription in response to an environmental cue. Either a ligand binding or protein-protein interactions can also play a role in regulation. Different sensory domains are present in an N-terminus of σ^54^—transcriptional regulators depending on an environmental signal to be detected [29]. In fact, MmoR proteins of *M. silvestris* BL2 and *M. stellata* AR4 are closely related to a group of σ^54^—transcriptional regulators that contain a GAF sensory domain. This group includes NifA, a regulator of nitrogen fixation, FhlA, a regulator of formate metabolism, and NorR regulator for NO detoxification [29]. The binding of 2-oxoglutarate to the GAF domain of NifA prevents the NifL-NifA protein interaction to ensure that NifA is not inhibited by NifL under nitrogen-fixing conditions [35]. FhlA contains two GAF domains that bind formate to activate the transcription of the formate hydrogen lyase system [36]. NorR contains a single GAF domain, which binds NO to activate transcription of *norVW* genes encoding flavorubredoxin and an associated flavoprotein, which reduce NO to nitrous oxide [37]. Our analysis of domain organization for MmoR proteins of *M. silvestris* and *M. stellata* revealed that the N-termini of both proteins contain a GAF domain not found in *M. trichosporium* OB3b or *M. capsulatus* Bath. Therefore, we cannot exclude the possibility that GAF domains of MmoR of either protein bind an unknown signal molecule to activate transcription of the *mmo* operon. However, even this doesn’t account for a different mode of regulation for the *mmo* genes. It is possible that MmoR is involved in protein-protein interactions with another protein to repress or activate transcriptional activation of *mmo* genes.

Using gel-shifts assays, Scanlan et al. [33] demonstrated that in order to bind upstream of the *mmo* promoter in *M. trichosporium* OB3b, MmoR requires a putative GroEL-like chaperon, MmoG. MmoG proteins are present and highly homologous in *M. silvestris* and *M. stellata*, and their presence or absence alone doesn’t explain a different mode of regulation. MmoG may be required to interact with another transcriptional regulator to adjust expression of the *mmo* operon in *M. silvestris* with respect to growth on different substrates.

Growth on methane is slower than on acetate or other multicarbon substrates for *M. silvestris* [17]. Methane is the least preferred substrate for *M. silvestris*. Because all methanotrophs demonstrate slow growth rates and the catalytic efficiencies (kcat/K_m_) of methane-oxidizing enzymes are lower than the median value of enzymes involved in central metabolism [38], it has been speculated that high levels of MMO expression could help methanotrophs to overcome limitations in carbon uptake. Therefore, even though *M. silvestris* cells grow more slowly on methane than on multicarbon substrates [17], high levels of sMMO expression partially compensate for that. *M. stellata* cells grow on methane more slowly than *M. silvestris* cells do, and indeed show lower levels of sMMO gene expression. An environmental cue that might enhance transcriptional activation of the *mmo* operon in *M. stellata* hasn’t been identified yet. We tested an influence of carbon dioxide (CO_2_) and cerium (III) on transcriptional activation of the *mmo* promoter in *M. stellata*. Neither CO_2_ nor cerium (III) could enhance transcriptional activation of the *mmo* promoter in *M. stellata*. It has been shown in *Methylosinus trichosporium* [26] and *Methylomicrobium buryatense* [28] that the addition of light REE (La, Ce, Pr, and Nd) upregulates the expression of a gene encoding XoxF methanol dehydrogenase and downregulates the expression of a gene coding for another type of methanol dehydrogenase, MxaF. Moreover, REE were essential as cofactor in the XoxF enzyme of the verrucomicrobial methanotroph, *Methylacidiphilum fumariolicum* SolV [27]. Farhan et al. [26] also suggested that in *Methylosinus trichosporium*, XoxF can only serve as the methanol dehydrogenase under conditions in which sMMO is expressed. In our experiments, the addition of cerium (III) did not affect the growth, methane consumption and *mmo* promoter activity in *M. stellata.* Therefore, a question about an environmental cue to enhance transcriptional activation of the *mmo* operon in *M. stellata* remains to be elucidated.

The most important issue, however, is still the question of what kind of transcriptional regulator(s) governs transcription of *mmo* genes in either *M. stellata* or *M. silvestris*, and why these differ so dramatically. A more detailed study is required to identify a potential regulator in each organism. In this study, we provide solid proof that a different mode of transcriptional regulation of sMMO contributes to the facultative lifestyle of *Methylocella silvetris* BL2 as compared to its sibling obligate methanotroph *Methyloferula stellata* AR4.

## Supplementary Materials

The following are available online at http://www.mdpi.com/2076-2607/6/1/20/s1. Figure S1. (a) Methane consumption by *M. silvestris* BL2 (open circles) and *M. stellata* AR4 (closed circles) cells. (b) Methane consumption by *M. silvestris* BL2 cells cultivated without addition of cerium (III) (closed triangles) and upon addition of 25 µM cerium (III) chloride (open triangles). Figure S2. Liquid cultures of *M. silvestris* BL2 cultivated without addition of cerium (III) (left) and upon addition of 25 µM cerium (III) chloride (right). Figure S3. Fluorescence microscopy images of *M. silvestris* BL2 and *M. stellata* AR4 cells carrying transcriptional fusions of a promoterless *gfp* to the respective *mmo* promoters and growing on either methane or acetate. Panels (a) and (b) show the *mmo* promoter of *M. silvestris* BL2 in *Methylocella*; panels (c), (d) show the *mmo* promoter of *M. stellata* AR4 in *Methylocella*. Figure S4. Quantitative estimation of the number of fluorescent GFP-expressing cells in *M. silvestris* (a) or *M. stellata* (b) above threshold in a flow cytometry analysis. *M. silvestris* or *M. stellata* cells carry transcriptional fusions of a promoterless *gfp* to either the *mmo* promoter of *M. silvestris* BL2, or the *mmo* promoter of *M. stellata* AR4. Cells carrying a *gfp* without any promoter fused to it were used as a control to determine threshold. Note that one replicate of a control sample grown on methanol was lost, leaving a single measurement. In the panel (b), the values for a control sample grown on methane were set to zero, since this value was used to define the threshold. Data are means ± 1SEM.

Supplementary File 1
