# Supplementary figures and images for: Differential Transcriptional Activation of Genes Encoding Soluble Methane Monooxygenase in a Facultative Versus an Obligate Methanotroph
Source: Microorganisms. 2018 Mar 6;6(1):20. doi: 10.3390/microorganisms6010020 (PMC5874634; doi:10.3390/microorganisms6010020)

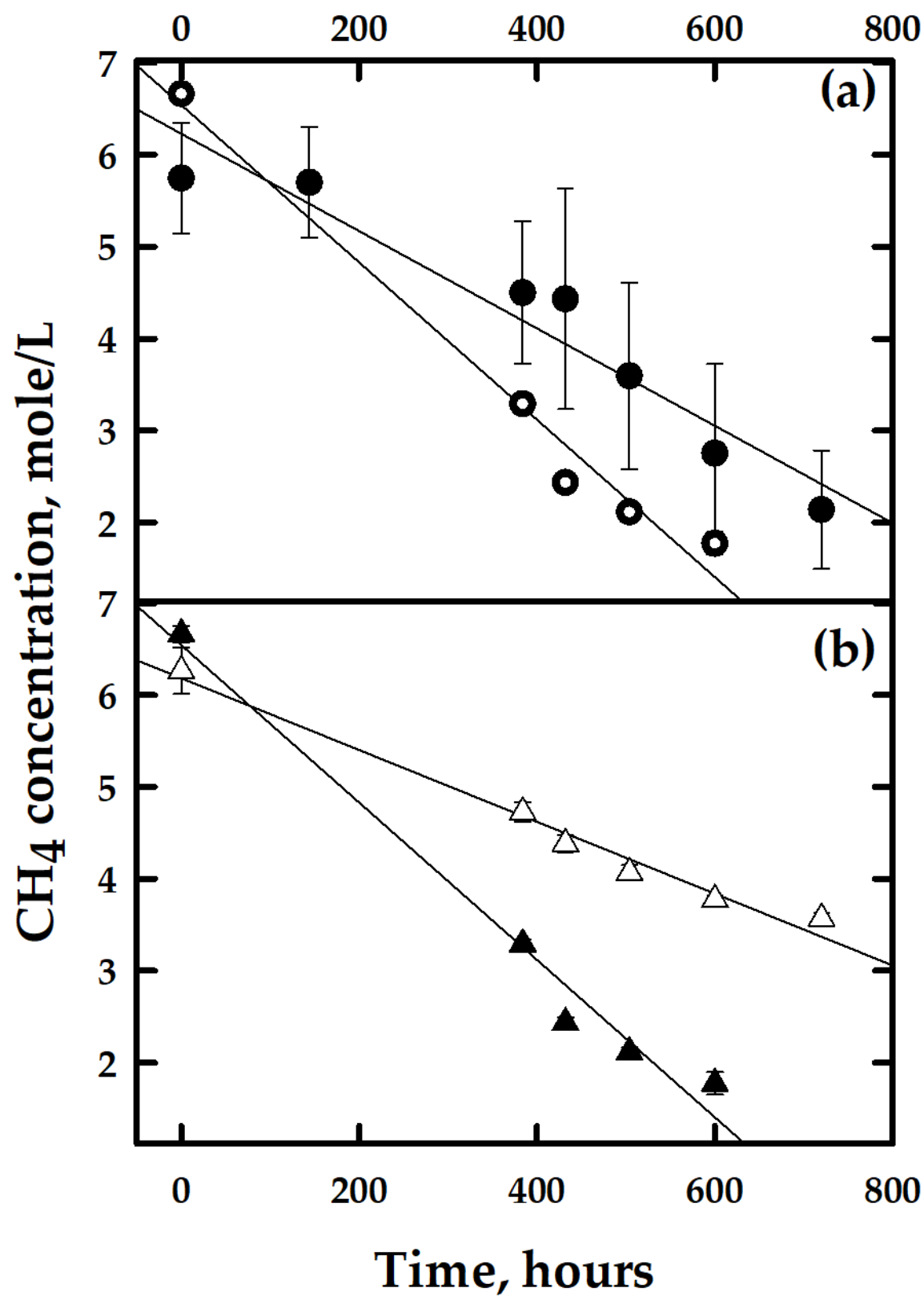

Supplement: Supplementary File 1 [file microorganisms-06-00020-s001.zip › Figures supplementary for Microorganisms ASmirnova PDunfield/Figure S1.PDF]

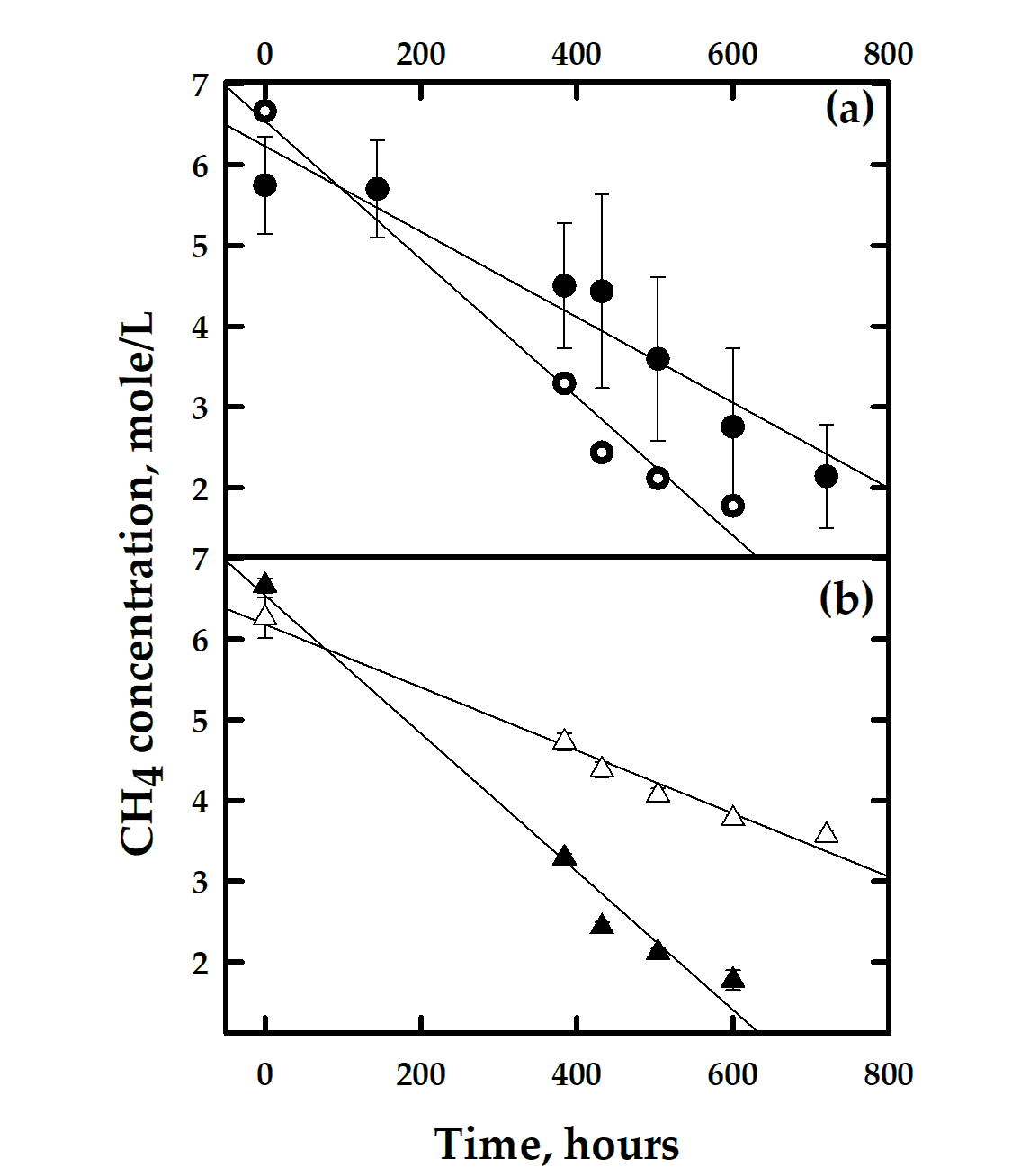

Supplement: Supplementary File 1 [file microorganisms-06-00020-s001.zip › Figures supplementary for Microorganisms ASmirnova PDunfield/Figure S1.TIF]

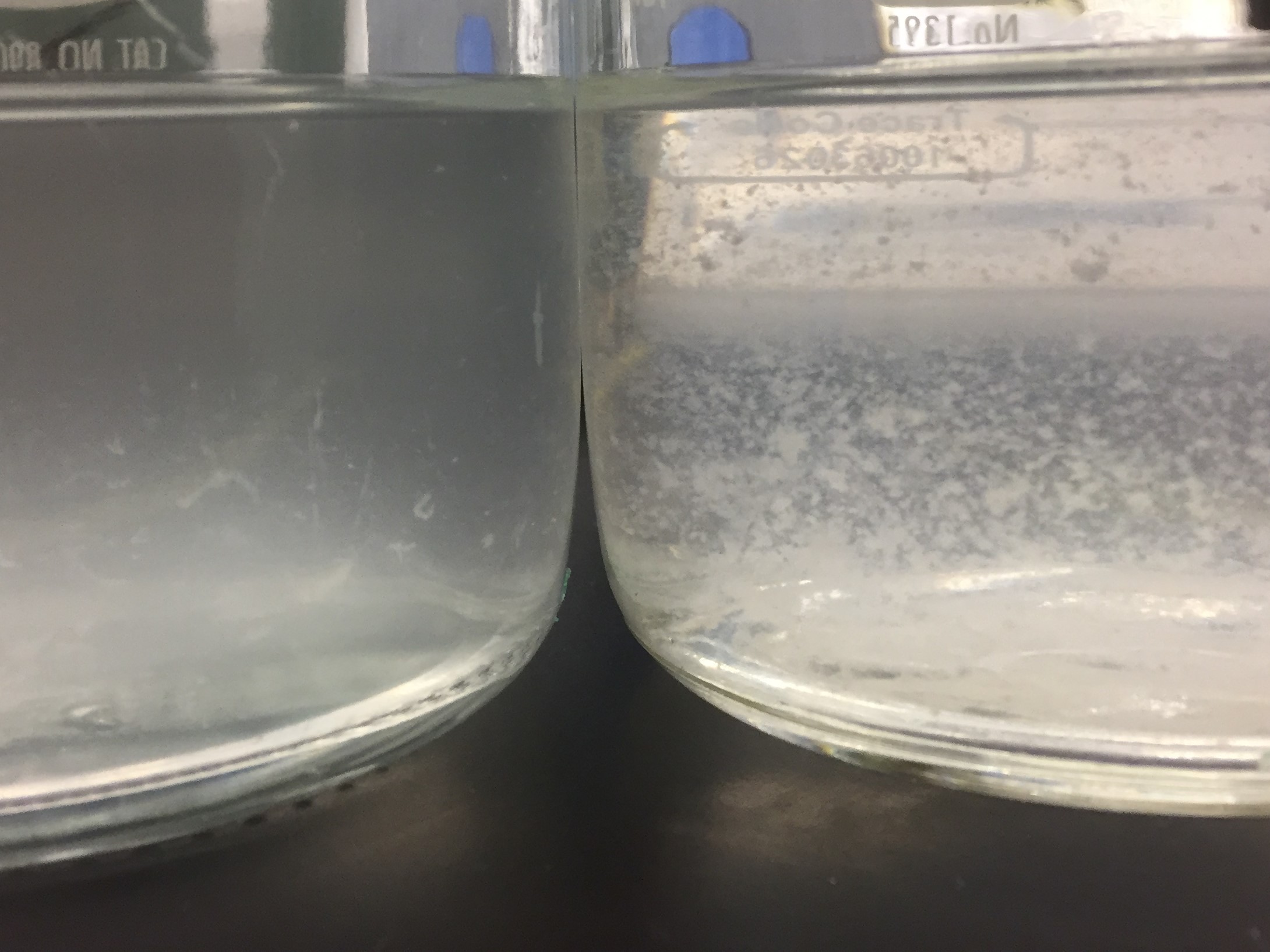

Supplement: Supplementary File 1 [file microorganisms-06-00020-s001.zip › Figures supplementary for Microorganisms ASmirnova PDunfield/Figure S2.JPG]

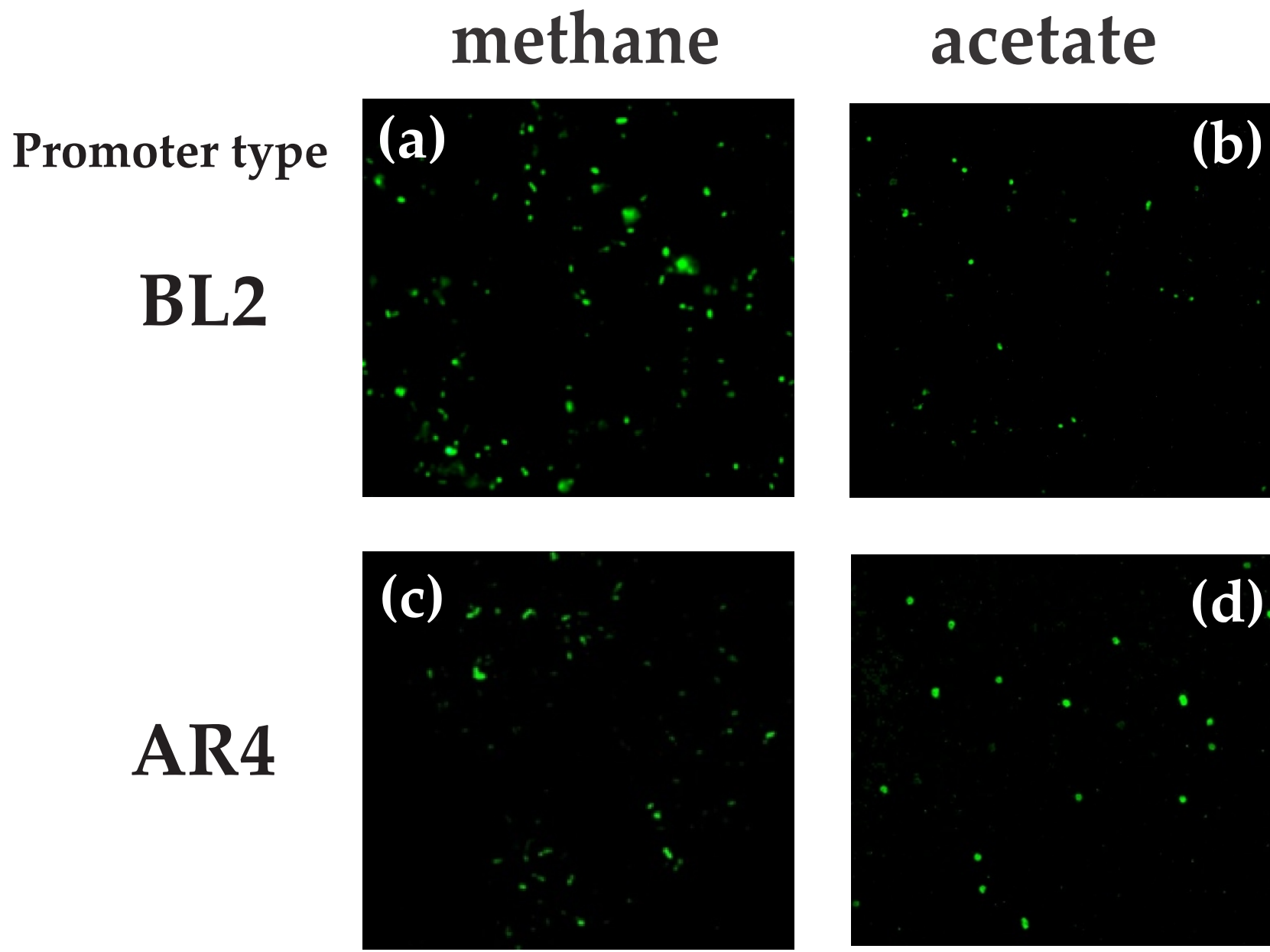

Promoter activity in  
*Methylocella silvestris* BL2

Supplement: Supplementary File 1 [file microorganisms-06-00020-s001.zip › Figures supplementary for Microorganisms ASmirnova PDunfield/Figure S3.pdf]

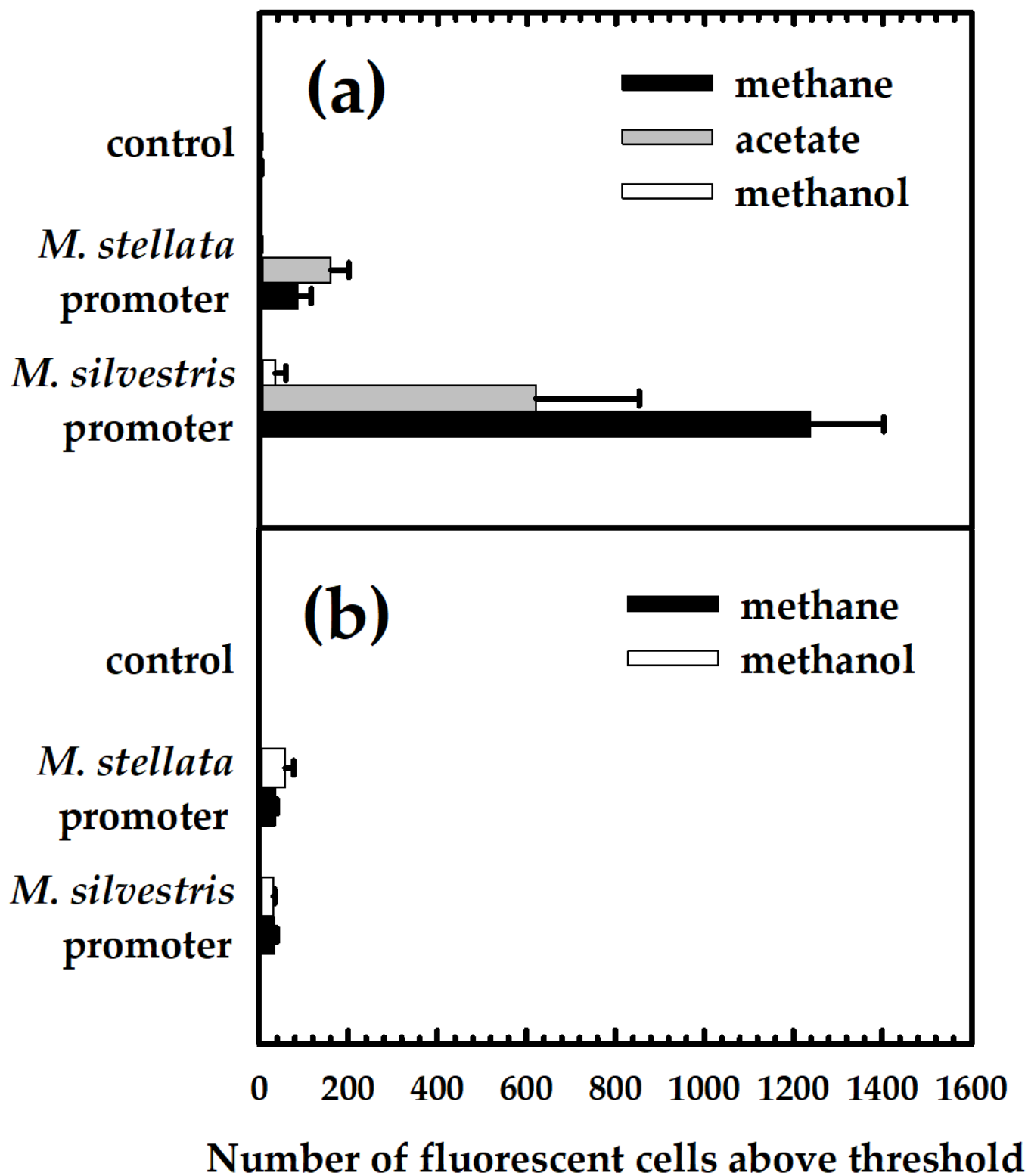

Supplement: Supplementary File 1 [file microorganisms-06-00020-s001.zip › Figures supplementary for Microorganisms ASmirnova PDunfield/Figure S4.pdf]
